# Evaluation of the Biological Effects of Lyophilized Hydrophilic Extract of* Rhus coriaria* on Myeloperoxidase (MPO) Activity, Wound Healing, and Microbial Infections of Skin Wound Tissues

**DOI:** 10.1155/2019/5861537

**Published:** 2019-07-14

**Authors:** Sami A. Gabr, Ahmad H. Alghadir

**Affiliations:** Rehabilitation Research Chair, College of Applied Medical Sciences, King Saud University, Riyadh, Saudi Arabia

## Abstract

Impaired wound healing was mainly associated with severe microbial infections which significantly affect diagnostic and therapeutic strategies. Thus, in this study, the potential wound healing activity, anti-inflammatory, and antimicrobial activity of an aqueous extract of* Rhus coriaria* extract (AERc) were evaluated by wound contraction, scar formation, period of epithelization, MPO enzyme activity, collagenase-2 (MMP-8), hydroxyproline (HPX), and collagen deposition as markers of wound healing at different days of postwound. Phytoconstituents, microbial activity, and fibrogenic markers were screened by HPLC, disc-diffusion, and colorimetric assays. The animals were treated with* Rhus coriaria* extract (AERc) concentrations at doses of 5 mg.kg^−1^and 10 mg.kg^−1^, respectively. On days 6 and 9, the AERc-treated animals at doses of 5 mg.mL^−1^ and 10 mg.mL^−1^ exhibited a significant reduction in the wound area, increased deposition of collagen, HPX, and reduction in MMP-8, and MPO enzyme activity when compared with controls. Scar formation and epithelization were completed in 10 days compared to controls. In addition, in wounds infected separately with* Staph. aureus or P. aeruginosa*, the AERc extract significantly improved wound contraction, deposition of collagen, and HPx and reduced MMP-8 and MPO concentrations, with complete epithelization of wounds in 10-13 days compared to the saline-treated group. Hydrolyzable tannins, gallic acid, quercetin, and myricetin were the most common active components of AERc.* In vitro*, the AERc and its components were effective against a set of microbes especially* Staph. aureus*,* P. aeruginosa*, and* Staph. aureus *(MRSA). In conclusion, the results showed that antimicrobial, anti-inflammatory, and antioxidant activity of* Rhus coriaria* extract suggested its importance as a target for formulation of novel drugs against many microbial infections with minimal side effects and could play a good potential role in accelerating wound healing activity via promoting myofibroblast activity, increase of hydroxyproline and collagen deposition, and regulation of MMP-8 and MPO enzyme activities.

## 1. Introduction

Wound healing is considered as one of the most complicated biological processes which is performed through a cascade of four overlapping phases: hemostasis, inflammatory, proliferative, and maturation. These stages result in the death of old tissues and regeneration of new ones. However, any defect or irregularity in this process may lead to severe microbial infections and impairment in wound healing, subsequently causing diagnostic and therapeutic problems [1, 2]. In infected wounds, the concomitant killing of regenerating cells was reported resulting in the formation of unpleasant exudates, toxins, and finally poor or lack of healing process[3, 4]. Up till now, drugs that could have efficient biological activities to promote and improve the natural process of wound healing with the human body, have not been introduced [5]. Consequently, to have a rapid healing process, we should prevent microbial infection and activate tissue repair processes [3].Thus, herbal medicines from natural sources have been shown to play an essential role in healthcare/promotion whereas about 80% of these plants have a globally ethnomedical use [6].

Among these herbal medicines is* Rhus coriaria L*. (Anacardiaceae), known as sumac, a wild edible-medicinal plant growing in tropical and temperate regions worldwide [7]. Sumac is one of the herbal plants traditionally used in the treatment of many diseases, including diabetes, cancer, and stroke [7–11]. Sumac was shown to have numerous health properties including antioxidant [12], antifibrogenic [13], antitumorigenic activities [14], and hypoglycaemic [15]. In addition, all parts of* R. coriaria* plants are known to possess nonmutagenic, fever reducing, DNA protective, antiseptic, antifungal, antibacterial, antioxidant, anti-ischemic, hypouricaemic, hypoglycaemic, and hepatoprotective properties, which support its traditional uses as wound healing agent particularly in infected wounds [11].

Myeloperoxidase (MPO) enzyme, is a haeme-containing protein secreted from Neutrophils as the first sign of inflammation or injury of cell and tissues [16–18]. In blood, MPO concentrations are currently measured as a marker of neutrophil initiation and degranulation [16–20]. Additionally, previous research studies showed that MPO concentrations are significantly lower in wound fluid (WF) of acute healing wounds compared to those present in tissues of pressure ulcers [21]. It is widely proposed that WF containing MPO before and after the healing process could have the potential to provide important biochemical information of wound healing and treatment against microbial infections [22, 23]. In monitoring the progression of wound healing, the MPO enzyme was shown to provide reliable diagnostic pieces of information about the overall status of a wound. In addition, wound exudates were used efficiently to follow up specified parameters which can help in diagnosis or in a particular wound therapy [23–26].

In this study, the wound healing activity and anti-inflammatory and antimicrobial activity of* Rhus coriaria* extract (sumac) were evaluated by wound contraction, scar formation, period of epithelization, MPO enzyme activity, collagenase-2 (MMP-8), hydroxyproline (HPX), and collagen deposition as markers of wound healing at different days of postwound.

## 2. Materials and Methods

### 2.1. Preparation of* Rhus coriaria* Aqueous Extracts

Fruits of* Rhus coriaria *were purchased from the local spice store (Othaim Markets) in Riyadh, Saudi Arabia. The dried whole fruits well-triturated in a homegrinder (Mx Type A505, Moulinex, Ecully Cedex, France). The aqueous extract of* Rhus coriaria,* called AERc, was prepared by infusing 50 g of powdered plant material for 20 minutes using 300 mL of boiling water. The extract was then filtered, lyophilized to produce a good yield of 23.4% (wt=wt). Upon use, the extract was reconstituted into the required concentration (5 mg.kg^−1^and 10 mg.kg^−1^) in sterile endotoxin-free water [1, 5].

### 2.2. Phytoconstituents Screening of* Rhus coriaria* Fruits Aqueous Extracts

The phytoconstituents present in aqueous extract of* Rhus coriaria *extract (AERc) were determined by using previously reported standard screening tests [26].

### 2.3. Assessment of the Total Anthocyanins Contents of Aqueous* Rhus coriaria* Extract

In the appropriately diluted* Rhus coriaria* aqueous extracts, the total concentrations of anthocyanins present were measured at 520 nm by a UV-Vis spectrophotometer (Varian Cary 100 Scan, Palo Alto, CA, USA) according to the pH differential method [27]. The total estimated values of anthocyanins were expressed as mg of cyanidin 3-glucoside equivalents (CGE)/kg of extract.

### 2.4. Assessment of the Total Phenolic and Flavonoid Contents of Aqueous* Rhus coriaria* Extract

The total phenolic content was determined by adding 5 mL of Folin–Ciocalteu (FC) commercial reagent diluted with water (1:10 v/v) and 4 mL of a 7.5% sodium carbonate solution as previously reported in the Folin–Ciocalteu (FC) colorimetric method [28]; then the mixture was stirred for 2 h at room temperature and kept away from strong light until a blue color appears. Finally, the total phenolic content was measured spectrophotometrically at absorbance of 765 nm, and the results were expressed as g of gallic acid equivalents (GAE)/kg of extract.

Similarly, the amounts of flavonoids in plant extracts were estimated according to the Dowd method as adapted by Lamien-Meda et al. [29]. The produced flavonoids were expressed as mg of Quercetin Equivalents (QE) /100 mg of fractions from the calibrated curve.

### 2.5. Estimation of Active Constituents in Aqueous* Rhus coriaria* Extract

Hydrolysable tannins, gallic acid, quercetin, and myricetin derivatives as active constituents were estimated in aqueous* Rhus coriaria* fruits extracts (AERc) by using HPLC method [29–32]. HPLC analysis using a 4.6 mm x 150 mm ODS C18 column with UV detector was performed to detect the bioactive phenolic compounds present in 50 mg of AERc water extract at significant mobile phase of methanol-dH2O (40: 60 %) adjusted at flow rate 0.60 ml/min and separation time of 15 min. The data were monitored using detector of 230 nm as previously reported [29–32]. These compounds were estimated in 23.4% (w/w) of the AERc product and were kept in the dark at −20°C until studied [11, 31, 33].

### 2.6. Antimicrobial Activity Test

Antimicrobial activities of AERc extract were evaluated against a set of microorganisms; gram positive* Staphylococcus aureus* (ATCC 12600),* Staphylococcus aureus *(MRSA), and* streptococcus aureus*. Gram negative* Pseudomonas aeruginosa* (ATCC 9721),* Escherichia coli* (ATCC 8677),* Proteus vulgaris*, and* Shigella spp*. (CIP 5451). In this study, to measure the antimicrobial activity of our AERc extract, the MRSA-strain was selected based upon the guidelines of the National Committee for Clinical Laboratory Standards [33]; the starin previously showed a resistance to methicillin (DMPPC) and oxacillin (MPIPC). Antimicrobial activity of AERc extract was estimated by previously reported modified diffusion test activity [34].

The colonies of the studied bacteria were picked directly from the plate and were suspended in 5 mL of sterile 0.85% saline. To have colony forming unites of 10^8^ (CFU)/ml, the turbidity of the initial suspension was adjusted by comparing with 0.5 McFarland's standard. All bacterial starins were grown in different petri dishes to exponential phase in Mueller-Hinton broth at 37°C for 18 h and compared with McFarland density [35]. The AERc extract at concentrations of 5 mg.kg^−1^ and 10 mg.kg^−1^ and blank and positive controls were added separately to each well (4 mm) of agar plate and allowed to diffuse at room temperature for 15-20 mim. The test was performed three times for each dose of AERc extract and positive control, respectively. In addition, chloramphenicol (1 mg.mL-1) and distillated water (40 *μ*L) were used as positive and blank controls respectively. Finally, after incubation for 24 hrs at 37°C, all plates were examined for growth inhibition zones and the diameter of each zone was measured. The average diameter in mm of the inhibition zone surrounding the wells containing the test solution was referred as the antimicrobial activity effects of AERc extract and positive control against microbial activity [1].

### 2.7. Animals

Fifty Wistar male rats of either sex weighing 180-250g were obtained from the experimental animal care center, college of applied medical sciences, King Saud Univ., Riyadh, Saudi Arabia. The animals were housed and subjected to normal feeding, drinking, and health care mechanism according to the guidelines of the breeding care unit at college of applied medical sciences. Animals had no history of surgery and infection, and other medical interventions were included in this study.

#### 2.7.1. Excision Wound Model Assessments

The animals were anesthetized with ketamine hydrochloride (50 mg/kg, i.p., body weight) in combination with xylazine hydrochloride (10 mg/kg) of body weight [36] and shaved at the predetermined area, dorsum portion using depilatory cream (Reckitt Benckiser, Inc., UK) beforehand wounding. A circular wound of approximately 2 cm in diameter was performed on the anterior-dorsal side of each mouse by using a sterile surgical blade as described previously [37].

### 2.8. Experimental Design

Based upon wound infection, the animals were randomly classified into two groups: non infected group (20 rats) and infected group (30 rats). In infected group, the wounds of the rats were inoculated (10 *μ*L) separately with* Staphylococcus aureus* (ATCC 25923) and* Pseudomonas aeruginosa* (ATCC 9721) at 10^8^ Colony Forming Unit (CFU). Wound infection model was estimated to be successful if yellowish white pus was present on the wounds. During topical wound treatments, the rats of both groups were further subdivided into the following groups as shown in Table 1

### 2.9. Wound Contraction and Epithelialization Assessments

Wound contraction and epithelialization period were calculated as percentage reduction in wound area. The progressive changes in wound area were monitored by a camera (Sony Cyber Shot, Dscw80) on wounding day, followed by measurements on 3rd,, 6th, and 9th day. Later on, the contraction in wound area was evaluated by using ImageJ program [37]. Following treatment periods, skin tissue samples from the heald sites were isolated from anesthetized animals and subjected for wound healing and histobiochemical analysis.

### 2.10. Assessment of Wound Healing and Epithelialization Rate

For each animal, the wound area was measured on days 3, 6, and 9 after surgery by tracing the wounds with the help of transparent sheet as previously reported [38]. The percent of the wound contraction was calculated from the data of the wound size measurements which was taken at the time of surgery and at the time of biopsy as previously reported [39], using the equation

(1) $$\begin{array}{c} \left[\;\frac{\left(\text{A0 – At}\right)}{A0}\;\right]\times 100=\%\text{ of wound closure} \end{array}$$

where A0 is the original wound area and At is the area of wound at the time of biopsy.

Epithelialization period was calculated as previously reported [40], as the number of days required for falling off the dead tissue remnants of the wound without any residual raw wound.

### 2.11. Assessment of Hydroxyproline

Hydroxyproline was estimated from a dried wound tissues at 60°C as previously reported [41]; the tissues were hydrolyzed with adding 5mL of 6N HCl for three hours at 130°C. Neutral hydrolysates (pH7.0) were subjected to Chloramine-T oxidation for 20 min in room temperature. After 10 min, 0.4 M perchloric acid was added as solution of stop reaction, termination of chloramine T oxidation. Finally for color development, 1 mL of Ehrlich's reagent was added to tubes, shook, and placed in water bath (60°C/20 min). Hydroxyproline concentration was measured in cooled solutions colorimetrically at 557nm by using ultraviolet (Systronics-2203) spectrophotometer. Hydroxyproline and collagen concentrations were measured according to the following equations:

(2) hydroxyproline  concentration g/ml=As - AbAst-Ab×concentration  of  standard 5g/ml×dilution  factor;  collagen  concentration g/ml=hydroxyproline  concentration×7.46×dilution  factor

see [42].

### 2.12. Assessment of Myeloperoxidase (MPO)

MPO activity was estimated in tissue samples as previously reported [43]. Briefly, 10 mL of wound tissue supernatant was mixed with 290 mL substrate solution, containing 100 mmol/L Guaiacol (Sigma-Aldrich) and 0.017% (w/w) H_2_O_2_ in 50 mmol/L potassium phosphate buffer (pH 7.0). For calibration, enzyme standards containing 0.16 U/mL up to 10.0 U/mL MPO (Sigma-Aldrich) were prepared. The resultant tetraguaiacol compound was estimated as measure of MPO activity every 20 s for 15 min at 470 nm. The change of optical density per minute was calculated five times from the initial rate. Finally, the increase in absorbance after 100 s was used for calculation of MPO activity. MPO activity was estimated in infected and noninfected wounds to determine the antioxidant and anti-inflammatory activity of AERc extract [43].

### 2.13. Assessment of Collagenase-2 (MMP-8)

Collagenase-2 (MMP-8) was estimated from the supernatant of excised wound tissues by enzyme immune assay ELISA technique as previously mentioned [44]. In ELISA plate, a total of 150 *μ*l of assay diluents RD1-52 was added and followed by the addition of 50 *μ*l of standard or sample tissues to each well. The palate was gently tapped to ensure thorough mixing, covered, and incubated for 2 h at room temperature (18-25°C). After incubation time, the plate was washed four times with washing buffer. To remove the excess washing buffer, the plate was inverted and blotted against clean paper towels and then a total of 200 *μ*l of MMP-8 conjugate was added to all the wells. The plate was then securely covered with a plate sealer and incubated for 1 h at room temperature. Again, the plate was washed 4 times followed by the addition of 200 *μ*l of substrate solution to each well and incubated for 30 min at room temperature out of the light. After incubation period (30 min), 50 *μ*l of stop solution was added in each well to stop the enzyme-substrate reaction. The optical density of the developed yellow color was determined within 30 min, using a microplate reader set to 450 nm as the primary wavelength. The levels of MMP-8 in the tissue samples were estimated using the standard curve and the concentrations were expressed as ng/ml.

### 2.14. Statistical Analysis

In this study, for the analysis of the data, a statistical software SPSS version 17 was used. The results obtained were expressed as Mean and standard deviation among groups; Kruskal–Wallis one-way ANOVA and and post-hoc (Tukey HSD) test were used to compare the mean values of the studied variables. Additionally, Spearman rank correlation analysis was performed to assess the relationship between various study parameters. The data obtained were deemed significant at P < 0.05.

## 3. Results

### 3.1. Phytoconstituents Screening

**Figure 1:**
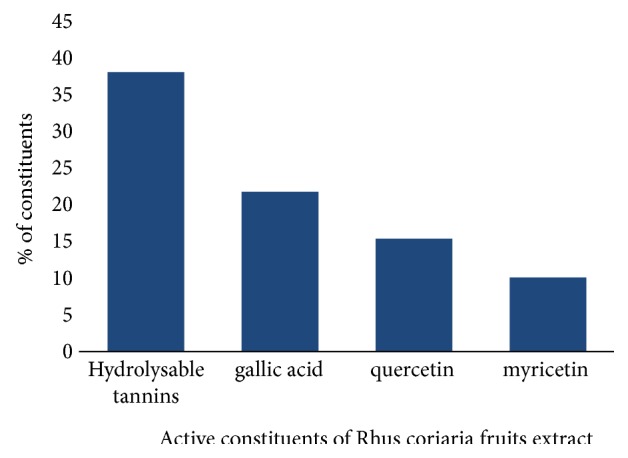
Screening of active Phytochemical contents present in aqueous extract of* Rhus coriaria *fruits.

A total of 23.4 % w/w of active phytoconstituents were estimated from* Rhus coriaria* fruits extracts (AERc). Alkaloids, flavonoids, glycosides, tannins, triterpenoids, glycosides, phenols, saponins, and anthraquinone were shown to be the most common phytoconstituents in AERc (Table 2). The aqueous extract of* Rhus coriaria* fruits was more concentrated in anthocyanins (98.3±0.21CGE/kg), flavonoids (78.6±0.81QE/g), and phenols (4.5±1.2 GAE/g). Hydrolyzable tannins (38.1%), gallic acid (21.8%), and quercetin (15.8%) were the most active components present in higher amounts, followed by lowest amounts of myricetin (10.1%) derivatives as active constituents present in AERc (Figure 1)

### 3.2. Antimicrobial Activity of* Rhus coriaria* Fruits Extracts

Table 3 shows the antibacterial activity of aqueous extracts of* Rhus coriaria* fruits against a set of bacterial strains;* Staph*. aureus,* P. aeruginosa, strep. aureus*,* E. coli*,* P. vulgaris*, and* Shigella spp*. The AERc at doses of 5 mg.mL^−1^ and 10 mg.mL^−1^ showed the highest antibacterial activity against* Staph*. aureus,* Staph*. aureus (MRSA),* Strep. aureus*, and* P. aeruginosa* compared to that present against* E. coli*,* P. vulgaris*, and* Shigella spp*. In addition, hydrolyzable tannins, gallic acid, quercetin, and myricetin were the most common active components that showed higher activity against the studied bacteria, while quercetin and myricetin at dose of 5 mg.mL^−1^ had no effect against* E. coli*,* P. vulgaris*, and* Shigella spp.,* respectively. Chloramphenicol, a standard antibiotic, showed significant antibacterial activity against the test organisms. Due to the potential higher activities of the AERc at doses of 5 mg.mL-1 and 10 mg.mL-1 against* Staph*. aureus ATCC 12600, the antimicrobial activity against* Staph*. aureus MRSA was assessed. Results demonstrated that the AERc at doses of 5 mg.mL-1 and 10 mg.mL-1 induced an inhibition zone of 19.7 and 22.4 mm for strain MRSA, value similar to that presented by the standard strain. Also, hydrolyzable tannins, gallic acid, quercetin, and myricetin showed a considerable inhibition activity against the growth of strain MRSA (Table 3).

### 3.3. Effect of AERc on Wound Closure and Epithelialization Period

**Figure 2:**
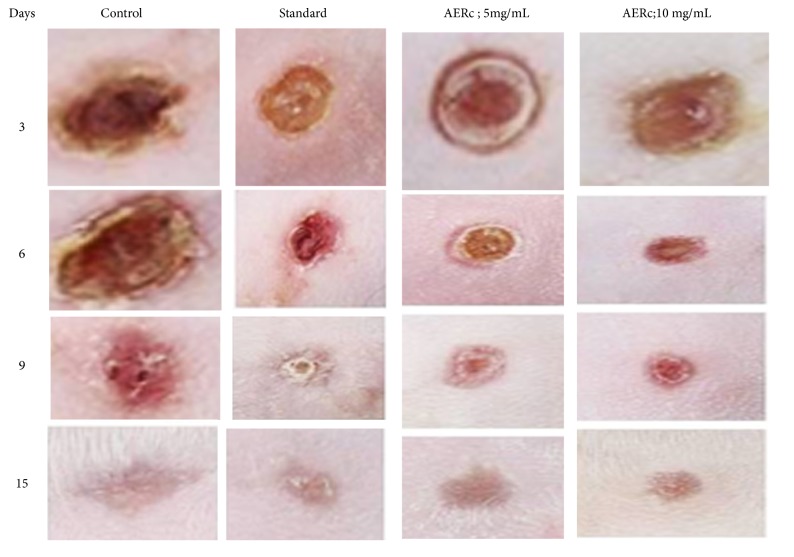
Photographs represent the percentage of wound closer rates on different postexcision days (3^th^ -* *-15^th^).

The wound healing activity of the aqueous extract prepared from* Rhus coriaria* fruits (AERc) was evaluated on mice in the excision wound models to confirm the potential healing and antimicrobial activity of the plant (Figure 2). In wounds treated with* Rhus coriaria* fruits extracts at doses of 5 mg.mL-1 and 10 mg.mL-1, the rates of wound closure were significantly increased from days 6^th^ to 15^th^ after wound excision compared to saline and standard treated rats, respectively (Figure 1).

In all groups, the area of the wound closure was measured on the days 3, 6 and 9 days after surgery. The progress of wound healing induced by the extract, reference drug, and saline-treated-groups in the excision of noninfected wounds are shown in Table 4.

The effect of AERc on the progression of wound closure, scar formation, and the period of epithelialization was estimated in noninfected wounds (Table 4). After application of AERc topically onto noninfected wounds, the area of wound reduced to 30%; 55% of their original size (2 cm^2^) on day 3, 57.5%; 77.5 % on day 6 and 83%; 91.5 on day 9; and complete closure around day 10 following treatment with AERc at doses of 5 mg.mL-1 and 10 mg.mL-1, respectively.

In saline-treated animals, the area was reduced to 12.0% (day 3), 22% cm^2^ (day 6), and 40.0% (day 9). The wound closure in animals treated with reference drug, Fibrase, was 15% (day3), 25% (day 6), and 54% (day 9) (Table 4). Treatment with AERc in noninfected wounds was able to reduce to 9 days the period of epithelialization when compared with the saline-treated group and Fibrase group, which were, respectively, 15.5 and 12.8 days.

Table 5 shows the changes in wound closure, scar formation, and the period of epithelialization in infected wounds treated with AERc. In wounds infected with gram-positive bacteria (*Staph*. aureus), the area of reduction in wounds was 30% and 40 % of their original size (2 cm2) on day 3, 40% and 52.% on day 6, and 96% and 97% on day 9, and complete closure on days 12 and 10 was reported following application of AEBv topically at doses of 5 mg.mL-1 and 10 mg.mL-1, respectively (Table 5).

In case of wounds infected with gram negative bacteria (*P. aeruginosa ), *there was significant reduction in wounds to 25% and 30 % of their original size (2 cm^2^) on day 3, 40% and 52.5 % on day 6, and 56.5% and 62.5% on day 9, and complete closure on days 13 and 12 was reported following application of AEBv topically at doses of 5 mg.mL-1 and 10 mg.mL-1, respectively (Table 4). On the other hand, in saline-treated animals, the area of wounds was reduced to 20 % (day 3), 30% (day 6), and 45% (day 9). The wound closure in animals treated with reference drug, Dermazine®, was 23.0% (day 3), 42%, and 62.5% (days 6 and 9) (Table 5). Treatment with AERc in infected wounds was able to reduce to around 10 -13 days the period of epithelialization when compared with the saline-treated group and Dermazine® group, which was, respectively, 19.5 and 16 days. In addition, complete scar formation occurred without any raw wound residues at days of complete epithelialization in noninfected wounds (9.5-10.8 days) and infected wounds (10-13 days) following AERC treatments at doses of 5 mg.mL-1 and 10 mg.mL-1, respectively. The mean scar area after complete healing was 98.5-99.8 mm^2^ for noninfected wounds and 94.7-99.8 mm^2^ for infected wounds, respectively (Tables 4 and 5).

### 3.4. Effect of AERc on Myeloperoxidase (MPO) Activity in Wound Tissues

**Figure 3:**
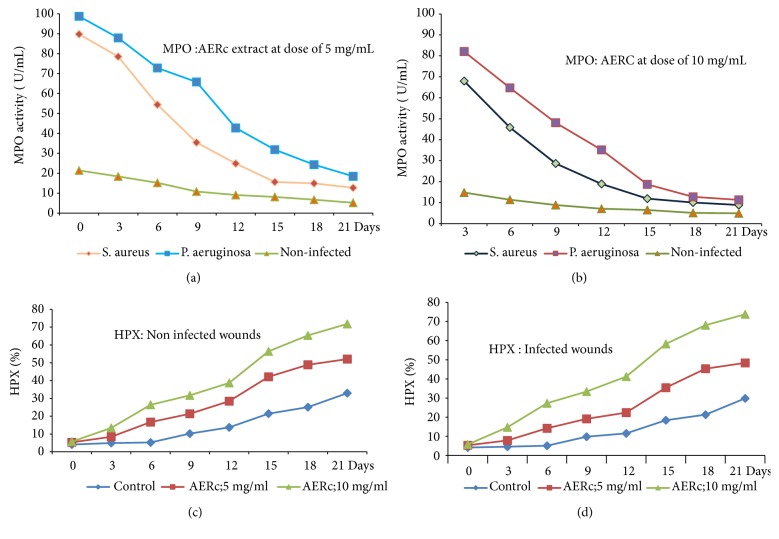
Effect of aqueous extract of* Rhus coriaria* fruits on myeloperoxidase (MPO) activity at a dose of 5 mg.mL^−1^ of AERc (a) and at adose of 10 mg.mL^−1^ of AERc (b) and hydroxyproline (HPX) in noninfected (c) and infected wounds (d) treated at 3, 6, 9, 15, and 21 postoperative days. In wounds infected with gram positive (*S. aureus*) and gram negative (*P. aeruginosa*), there was significant decrease (p=0.001) in the level of MPO activity and increase (p<0.05) in the levels of HPX at days 15th, 18^th^, and 21^st^ following treatment with AERc at doses of 5 mg.mL^−1^, 10 mg.mL^−1^, and compared with control treatments, respectively.

MPO enzyme activity was determined as a potential marker for the diagnosis of wound infection and healing process in skin wounded tissues infected separately with gram-positive and gram-negative bacteria. In this study, significantly higher MPO activity was detected in infected skin wounds (*P. aeruginosa or Staph*. aureus) when compared with noninfected wounds based on the oxidation of guaiacol using commercial MPO as standard (Figures 3(a) and 3(b)). After application of AERc topically at doses of 5 mg.mL-1 and 10 mg.mL-1 onto infected and noninfected wounds, the levels of MPO activity decreased significantly at 9, 12, 15, 18, 21 days after surgery in all groups (Figures 3(a) and 3(b)).

The decrease in MPO activity was negatively correlated (P=0.001) with wound closer, scar formation, and complete epithelialization following treatments with AERc at the recommended doses (Table 6). The reduction in MPO activity greatly supports the antimicrobial and wound healing activities of* Rhus coriaria* fruits (AERc) against infection of skin wound tissues with gram-positive and gram-negative bacteria.

### 3.5. Effect of AERc on Fibrogenesis Markers

**Figure 4:**
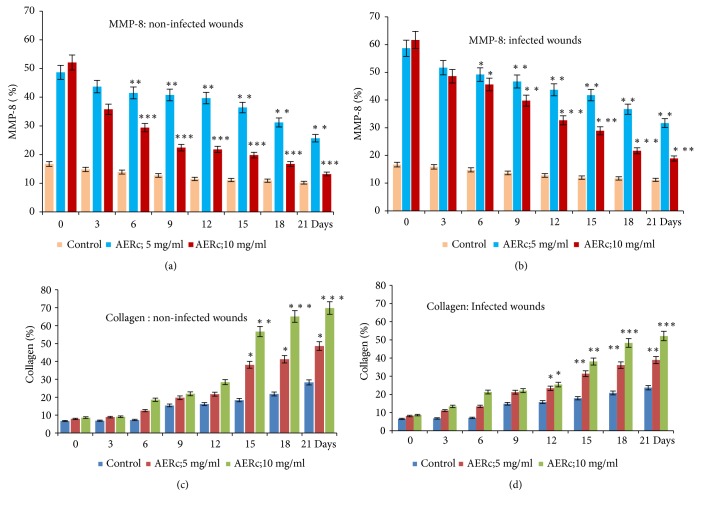
Mean values and standard errors of the percentage of MMP-8 (a) in noninfected, (b) infected wounds, and collagen in noninfected (c), and infected wounds (d). Wounds are infected separately with 10 *μ*L* of S. aureus* (ATCC 25923) and* P. aeruginosa *at 108 Colony Forming Unit (CFU). The data showed significant depletion in the activity of MMP-8 and increase in the level of collagen deposition in wounds of control and treated animals at 3, 6, 9, 15, and 21 postoperative days.

The levels of HPX and collagen were significantly (*P* < 0.001) higher in AERc, Dermazine, and fibrase treated rats than they were in saline-treated rats (control). Moreover, it was observed that the effects of AERc and standard controls (Dermazine and fibrase) were dose-dependent (Table 6). Compared to the normal control rats, both infected and noninfected skin wounds showed a significant increase (*P* < 0.001) in the levels of HPX and collagen as the fibrogenesis markers in their wounds (Table 6). In infected and noninfected skin wounds, a significant increase in the levels of HPX and collagen was observed at days 6, 9, 12, 15, 18, and 21 after treatment with AERc at doses of 5 mg.mL-1 and 10 mg.mL-1 as shown in Figures 3(c), 3(d), 4(c), and 4(d).

MMP-8 as a fibrogenic potential marker for measuring active wound healing was also estimated in tissue samples of noninfected and infected skin wounds (Table 6). In groups treated with AERc at doses of 5 mg.mL-1 and 10 mg.mL-1, the levels of MMP-8 activity greatly reduced in comparison with standard and saline-treated groups (*P* < 0.001). The decrease in the levels of MMP-8 starts at days 6 up to 21 after treatment with AERc recommended doses and it showed to be significantly associated with the activity of AERc extract towards the acceleration of wound healing process in both infected and noninfected skin wounds as shown in Figures 4(a) and 4(b).

The expression rates of HPX, collagen deposition, and MMP-8 activity in skin wounds following treatment with AERc at doses of 5 mg.mL^−1^ and 10 mg.mL^−1^ showed significant correlation with wound healing parameters (Table 7). The rate of the wound closure, scar formation, and complete epithelialization correlated positively (*P* < 0.001) with HPX, collagen deposition, and negatively (*P* < 0.001) with MMP-8 activity following treatment with the recommended doses of AERc extract.

## 4. Discussion

Topical application of an aqueous extract of* Rhus coriaria* fruits (AERc) on infected and noninfected skin wounds of rats has shown the efficacy of the extract (AERc) in facilitating wound healing process at doses of 5 mg.mL-1 and 10 mg.mL-1, respectively. Additionally, wound healing promotion activity of AERc extract has been evaluated based on our screening analysis of its active phytoconstituents. The results showed that the aqueous extract (AERc) contains sufficient amounts of alkaloids, flavonoids, glycosides, tannins, triterpenoids, glycosides, phenols, saponins, and anthraquinone whereas anthocyanins and flavonoids were estimated in higher amounts in AERc extract followed by a considerable amount of phenols. The most active constituents present in the extract were hydrolyzable tannins, gallic acid, quercetin, and myricetin derivatives as measured by HPLC.

The presence of these active constituents was shown to be responsible for numerous health properties of* Rhus coriaria* fruits in treating many diseases as antidiabetes, anticancer, and antistroke [7–11]. Also, sumac was used traditionally in treating many physiological and cellular disorders as an antioxidant [12], antifibrogenic [13], antitumorigenic activities [14] and hypoglycaemic [15], DNA, and hepatoprotective properties [11]. So, due to the potential biological activities previously mentioned for* Rhus coriaria fruits* extracts, it can be suggested that this plant may have greater efficiency to promote wound healing and contribute in skin regeneration of infected and noninfected wounds.

In this study, the topical application of* Rhus coriaria fruits* extracts (AERc) at doses of 5 mg.mL-1 and 10 mg.mL-1 enhances cutaneous wound healing, which appeared completed in 10 days for noninfected skin wounds and around 10-13 days for infected wounds according to the type of microbes and used dose of* Rhus coriaria fruits* extracts.

The histochemical findings showed that wound healing, scar formation, and the epithelialization rates, as well as tissue regeneration, were significantly correlated with greater expression of hydroxyproline, deposition of collagen fibers, and lower in the activities of both MMP-8 and MPO in skin wounds treated with the extract than in saline-treated wounds. The changes in fibrogenic markers for improving the healing process were mediated by myofibroblasts described firstly in healing skin wounds. It was proposed that myofibroblasts were responsible for the phenomenon of wound contraction [45, 46].

In granulated wound tissues, fibroblasts are activated to become myofibroblasts via acquiring *α*-SM-actin expression. During the wound healing process, the activated myofibroblastic cells synthesize and deposit the surrounding extracellular matrix (ECM) components such as collagen and hydroxyproline, control the activity of MMP-8 and MPO, and eventually replace the provisional matrix. Additionally, it was reported that during healing, scar formation involves a progressive remodeling of the granulation tissue via expression of proteolytic enzymes, essentially matrix metalloproteinases (MMPs) particularly our MMP-8 enzyme one of these enzymes which play a major role with their inhibitors (tissue inhibitor of metalloproteinases [TIMPs]) in wound healing [47]. Also, it was reported that during healing of wounds, the deposited collagen fibers are changed from collagen type III, the major component of the granulation tissue to collagen type I which is the main structural component of the dermis, followed by elastin, which contributes to the final stage of skin epithelialization and elasticity. In addition, successive stages of synthesis, degradation, and orientation of collagen fibrils were involved in the scar remodeling process. During scar formation, collagen is deposited and randomly oriented to fibronectin fibers oriented in such manner depending on the nature and direction of the tensional forces applied to the scar [48–50]. At the final of healing, the cell number vascular cells and myofibroblasts are dramatically reduced by apoptosis [51]. So, the increase in wound contraction in AERc-treated rats might be a result of the enhanced activity of fibroblasts.

In this study, the increase in the activity of MMP-8 and MPO was the first response to inflammation which starts immediately after wounding process, acting as a defense mechanism of the tissue, able to provide resistance to the microbial contaminations [52]. The release of MPO in tissues and blood circulation result from inflammatory processes [53, 54]. Thus it considered now as a marker of neutrophil activation and degranulation [19, 55].

Gutiérrez-Fernández et al.[20] reported that matrix metalloproteinases (MMPs) have been implicated in numerous tissue-remodeling processes, especially collagenase-2 (MMP-8). They found that mice deficient in collagenase-2 (MMP-8) are more susceptible to develop skin cancer and delay in wound healing. The data of this study proved that the presence or expression of MMP-8 participates in wound repair by contributing to the resolution of inflammation stage.

In our study, the reduction in the activity of MMP-8 and MPO appeared at days 6-9 after surgery. This may be related to the progression of the proliferation and maturation phases of wound healing following treatment with AERc. So, the improved healing process might be due to the anti-inflammatory activity of AERc extract whereas the wound healing process significantly delayed with long durations in the inflammatory phase [20]. Also, it was shown that the anti-inflammatory activity of the extract is essential for better wound healing in shorter periods [56]. Therefore, as previously reported the activity of our AERc extract was significantly related to its anti-inflammatory effects [57, 58]. Also, the healing activity of sumac was shown to play a role in decreasing MPO enzyme activity [5]. These data provide evidence that* Rhus coriaria* extract accelerates cutaneous wound healing through decreasing MPO activity and oxidative damage in both infected and noninfected skin wounds. Previously, it was reported that Sumac leaves are good sources of phenolic acids and several flavonoids used frequently against many diseases as anti-inflammatory, antioxidant, antibacterial, fungicide, antiviral, and candidicide [59, 60].

A delay in the wound healing process was mainly associated with microbial attacks for wounds exposed to external unfavorable environments which may compromise the repair process. In skin wounds,* Staph*. aureus and* P. aeruginosa *are the most common pathogens responsible for infection [61, 62]. Topical application of the extract AERc and standard drugs are more effective as a microbicide and increasing wound healing rate in a shorter time compared to control saline-treated rats. This is may be due to its greater availability at the infected wound site, compromise inflammation, and lesions produced due to microbial infections. In control rats, the slow rate of wound contraction is controlled by the presence of microorganisms which secrete metabolites, inhibiting wound contraction, and subsequently impair healing.

In infected wounds, even the period of epithelialization is greater compared with noninfected animals treated with AERc at doses of 5 mg.mL^−1^ and 10 mg.mL^−1^; there was a better wound healing if compared to animals treated with saline. The data was supported by previous research work which confirmed the extract of Sumac (*Rhus coriaria*) is used in traditional medicine as a medicinal herb for its antimicrobial and wound healing activity [5, 7]

Also, to confirm the potential role of AERc sumac extract as antimicrobial in the healing process,* in vitro *analysis of the antimicrobial effect of AERc was performed against a set of gram-positive and gram-negative microorganisms. The data showed that AERc has a significant potential inhibitory effect against* Staph*. aureus,* P. aeruginosa, Strep. aureus*,* E. coli*,* P. vulgaris*, and* Shigella spp*.

In addition, hydrolyzable tannins, gallic acid, quercetin, and myricetin were the most common active components that showed higher activity against the studied bacteria, except for the fact that quercetin and myricetin had no effect against* E. coli*,* P. vulgaris*, and* Shigella spp.* at lower dose (5 mg.mL-1). In our previously reported studies, whole* Rhus coriaria* fruits extract and their active constituents showed higher antioxidant and antimicrobial activity against bacteria and fungi [63–65] Furthermore, our study showed that AERc was effective against a methicillin-resistant strain of* Staph*. aureus (MRSA). It was reported that MRSA are multiresistant and difficult for treatment due to the fact that up till now there are no satisfactory antimicrobial drugs [1, 33]. Therefore, regarding our present result, extract from* AERc *seem to have a potential efficiency to combat the problem of MRSA.

In conclusion, the results showed that antimicrobial, anti-inflammatory, and antioxidant activity of* Rhus coriaria* extract suggested its importance as a target for formulation of novel drugs against many microbial infections with minimal side effects and could play a good potential role in accelerating wound healing activity via promoting myofibroblast activity, increase of hydroxyproline and collagen deposition, and regulation of MMP-8 and MPO enzyme activities.

## Figures and Tables

**Table 1:** Full elucidation of experimental design.

Group	Description
G1:	Saline non infected group; rats received topical application (200 *μ*L) of solutions containing saline (NaCl, 0.9%).

G2:	Standard treatment group; non-infected rats received fibrinolysin (Fibrase SA®) topically as once daily at a dose of 0.5 g.

G3:	Non infected Rats treated with AERc extract at doses of 5 mg.kg-1 for once a day for 9 consecutive days starting from the day of wounding.

G4:	Non-infected Rats treated with AERc extract at doses of 10nmg.kg-1 for once a day for 9 consecutive days starting from the day of wounding.

G5:	Saline infected group; infected rats with either *Staphylococcus aureus* (ATCC 25923) or *Pseudomonas aeruginosa* received topical application (200 *μ*L) of solutions containing saline (NaCl, 0.9%).

G6:	Infected rats with either *Staphylococcus aureus* (ATCC 25923) or *Pseudomonas aeruginosa* received 1% silver sulfadiazine (Dermazine®) topically as standard treatment once daily

G7:	Infected rats with *Staphylococcus aureus* (ATCC 25923) treated with AERc extract at doses of 5 mg.kg-1 for once a day for 9 consecutive days starting from the day of wounding

G8:	Infected rats with *Staphylococcus aureus* (ATCC 25923) treated with AERc extract at doses of 10 mg.kg-1 for once a day for 9 consecutive days starting from the day of wounding

G9:	Infected rats with *Pseudomonas aeruginosa* received AERc extract at doses of 5 mg.kg-1 for once a day for 9 consecutive days starting from the day of wounding

G10:	Infected rats with *Pseudomonas aeruginosa* received AERc extract at doses of 10 mg.kg-1 for once a day for 9 consecutive days starting from the day of wounding

**Table 2:** Phytoconstituents screening of aqueous Rhus coriaria fruits extracts (AERc mg/ 50mg).

Item	AERc mg/ 50mg
Percentage yield	23.4 %
*Phytochemical screening (+/-):*	
Alkaloids	+
Flavonoids	+
Tannins	+
Glycosides	+
Triterpenoids	+
phenols	+
Saponins	+
Quinone	-
Anthraquinone	+
*Phytochemical constituents (M ± SD)*	
Total phenolics ^1^	4.5± 1.2
Total flavonoids ^2^	78.6± 0.81
Total Anthocyanins ^3^	98.3± 0.21

(+/-) presence or absence of phytoconstituents; phytochemical constituents represented as mean ± SD (*n* = 3).  ^1^ Expressed as mg of gallic acid equivalents (GAE)/g of the dry extract.  ^2^ Expressed as mg of quercetin equivalents (QE)/g of the dry extract.  ^3^ Expressed as mg of cyanidin 3-glucoside equivalents (CGE)/kg.

**Table 3:** Antibacterial activity of the aqueous *Rhus coriaria* fruits extracts and active phytoconstituents.

Treatment	Dose (mg.mL^−1^)	Zone of inhibition (mm)						
		*Staph. aureus*	*Staph. aureus (MRSA)*	*Strepto. aureus*	*P. aeruginosa*	*E. coli*	*P. vulgaris*	*Shigella spp.*
Chloramphenicol	1	20.8±0.01	18.8±0.0	19.2±0.0	12.8±0.01	11.8±0.0	12.1±0.0	13.6±0.0
AERc	5	19.8±0.01	19.7±0.01	17.6±0.0	13.7±0.0	11.1±0.0	10.8±0.0	12.9±0.01
	10	22.4±0.0	22.4±0.0	18.4±0.0	14.4±0.0	12.7±0.0	11.9±0.0	13.0±0.0
Tannin	5	15.1±0.0	15.7±0.0	14.8±0.0	14.1±0.0	15.1±0.0	11.1±0.0	10.6±0.0
	10	16.6±0.0	16.1±0.0	15.3±0.0	15.9±0.0	13.1±0.0	12.2±0.0	12.6±0.0
Gallic acid	5	17.8±0.0	16.9±0.0	15.7±0.0	14.1±0.0	13.7±0.0	11.8±0.0	13.7±0.0
	10	19.6±0.0	18.9±0.0	17.6±0.0	13.2±0.0	11.2±0.0	10.2±0.0	9.8±0.0
Quercetin	5	16.8±0.0	15.8±0.0	14.1±0.0	10.1±0.0	NA	NA	NA
	10	17.9±0.0	16.3±0.0	15.9±0.0	12.6±0.0	11.7±0.0	11.0±0.0	10.2±0.0
Myricetin	5	15.1±0.0	14.8±0.0	13.1±0.0	9.1±0.0	NA	NA	NA
	10	16.6±0.0	15.3±0.0	15.6±0.0	11.5±0.0	8.2±0.0	6.2±0.0	7.3±0.0

NA, not active. Values represent the mean±SD. *Staphylococcus aureus* (ATCC 12600), *streptococcus aureus*, gram negative *Pseudomonas aeruginosa* (ATCC 9721), *Escherichia coli* (ATCC 8677), *proteus vulgaris*, *shigella spp*. (CIP 5451), and *Staphylococcus aureus* (MRSA).

**Table 4:** Effect of aqueous extract of Rhus coriaria fruits on non-infected wounds.

Treatment	Parameters of non-infected wounds				
	Wound area (cm^2^) on day			Scar area (mm^2^)	Period of epithelization (Days)
	3	6	9		
G1	1.76 ± 0.16	1.56 ± 0.12	1.2 ± 0.09	90.8± 3.4	15.5± 0.5
G2	1.7 ± 0.25	1.5 ± 0.10	0.92 ± 0.07 ^a^	97.1± 2.5 ^b^	12.8± 0.65 ^a^
AERc					
G3 (5 mg.mL^−1^)	1.4 ± 0.15	0.85 ± 0.06	0.34 ± 0.04 ^b,c^	98.5± 1.3 ^b,c^	10.8± 0.6 ^b,c^
G4 (10 mg.m)	1.1 ± 0.07	0.45 ± 0.04	0.17 ± 0.02 ^a,c^	99.8± 1.1 ^a,c^	9.5± 0.32 ^a,c^

Values represent the mean ± SD, (n=10). Statistical differences were determined by ANOVA followed Student Newman-Keuls test. G1: rats with non infected wounds treated topically with saline solution (200 *μ*L of NaCl, 0.9%); G2: rats with non infected wounds treated with Fibrase® topically at dose of 0.5 g; G3: rats with non infected wounds treated topically with AERc at dose of 5 mg.mL^−1^; G4: rats with non infected wounds treated topically with AERc at dose of 10 mg.mL-1.  ^a^ P<0.001,  ^b^ P<0.01, as compared to respective saline treatment (G1).  ^c^ P<0.001 as compared to Fibrase-treated animals (G2). AERc: aqueous extract of *Rhus Coriaria* extract.

**Table 5:** Effect of aqueous extract of *Rhus coriaria* fruits on infected wounds.

Treatment	Wounds infected with Gram positive (*Staph. aureus*)				
	Wound area (cm^2^) on day			Scar area (mm^2^)	Period of epithelization (Days)
	3	6	9		
G5	1.6 ± 0.06	1.4 ± 0.06	1.1 ± 0.06	91.8± 1.4	19.5± 0.6
G6	1.54 ± 0.04	1.16 ± 0.03	0.75 ± 0.05 ^a^	97.3± 3.5 ^b^	16.0± 0.0 ^a^
AERc					
G7 (5 mg.mL^−1^)	1.4± 0.12	0.58 ± 0.08	0.08 ± 0.03 ^b,c^	98.7± 1.9 ^b,c^	12.0± 0.0 ^b,c^
G8 (10 mg.mL^−1^)	1.2± 0.11	0.35 ± 0.02	0.06 ± 0.01 ^a,c^	99.8± 0.4 ^a,c^	10.8± 0.1 ^a,c^

Treatment	Wounds infected with Gram Negative (P. *aeruginosa*)				
	Wound area (cm^2^) on day			Scar area (mm^2^)	Period of epithelization (Days)
	3	6	9		

AERc					
G9 (5 mg.mL^−1^)	1.5± 0.19	1.2 ± 0.11	0.87 ± 0.21 ^b,c^	94.7± 2.9 ^b,c^	13.0± 0.0 ^b,c^
G10 ( 10 mg.mL^−1^)	1.4± 0.18	0.95 ± 0.12	0.75 ± 0.10 ^a,c^	99.1± 0.4 ^a,c^	12.0± 0.0 ^a,c^

Values represent the mean ± SD (n=10). Statistical differences were determined by ANOVA followed Student-Newman-Keuls test. G5: infected rats with either *Staphylococcus aureus* (ATCC 25923) or *Pseudomonas aeruginosa* received topical application (200 *μ*L) of solutions containing saline (NaCl, 0.9%); G6: infected rats with either *Staphylococcus aureus* (ATCC 25923) or *Pseudomonas aeruginosa* received 1% silver sulfadiazine (Dermazine®) topically as standard treatment once daily; G7: infected rats with *Staphylococcus aureus* (ATCC 25923) treated with AERc extract at doses of 5 mg.kg-1 for once a day for 9 consecutive days; G8: infected rats with *Staphylococcus aureus* (ATCC 25923) treated with AERc extract at doses of 10 mg.kg-1 for once a day for 9 consecutive days; G9: infected rats with *Pseudomonas aeruginosa* received AERc extract at doses of 5 mg.kg-1 for once a day for 9 consecutive days; G10: infected rats with *Pseudomonas aeruginosa* received AERc extract at doses of 10 mg.kg-1 for once a day for 9 consecutive days.^a^  P<0.001, ^b^  P<0.01 as compared to respective saline treatment. ^c^  P<0.001 as compared to Dermazine-treated animals.

**Table 6:** Effect of aqueous extract of *Rhus coriaria* fruits on collagenase-2 (MMP-8), hydroxyproline (HPX), and collagen contents in infected and non-infected excision wounds.

Treatment	Wounds					
	Non-infected			Infected		
	MMP-8	Collagen	HPX	MMP-8	Collagen	HPX
Saline	48.9 ±8.5	5.4 ±0.47	115.4 ± 3.4	75.1±12.5	3.5 ±0.53	85.4 ± 2.9
Fibrase®	45.6 ±5.6 ^a^	5.4 ±0.51	121± 6.4 ^a^	71.3±6.5 ^a^	3.6 ±0.35 ^a^	89.1 ± 3.1 ^c^
Dermazine®	41.3 ±4.7 ^b^	5.1±0.43 ^a^	118.7 ± 7.1 ^a^	63.9±7.4 ^b^	6.1 ±0.58 ^b^	135 ± 5.4 ^c^
AERc						
5 mg.mL^−1^	31.8 ±2.8 ^c^	11.6±0.31 ^c^	181.4 ± 5.7 ^c^	58.9±4.9 ^c^	10.2 ±0.72 ^c^	169.8 ±2.1 ^c^
10 mg.mL^−1^	21.3 ±1.2 ^c^	19.6±0.27 ^c^	215.8 ± 9.1 ^c^	44 ±6.1 ^c^	15.7 ±0.35 ^c^	269 ± 2.6 ^c^

Values are mean ± SD of 10 rats in each group. ^a^*P* < 0.05, ^b^*P* < 0.01, and ^c^*P* < 0.001 compared to respective control groups (Fibrase® and Dermazine®) and standard. Statistical analysis was done by one-way analysis of variance followed by Tukey-Kramer Multiple Comparisons Test.

**Table 7:** Correlation between parameters of wound closer and collagenase-2 (MMP-8), Myeloperoxidase (MPO) activity, hydroxyproline (HPX), and collagen contents in infected and non-infected wounds treated with aqueous extract of *Rhus coriaria* fruits.

Item	wound closer (%)	Scar formation (mm2)	Epithelialization period (Days)
Hydroxyproline (*μ*g/g of tissues)	0.14 ^b^	0.42 ^b^	0.28 ^b^
collagen(g/mL)	0.78 ^b^	0.58 ^b^	0.85 ^b^
MPO activity(U/mL)	-0.45 ^b^	-041 ^b^	-0.47 ^b^
MMP-8 (ng/ml)	-0.23 ^b^	-0.65 ^b^	-0.54 ^b^

Data presented as coefficient (*R*); ^a^  significance at <0.01; ^b^  significance at <0.001.

## Data Availability

All data generated or analyzed during this study are presented in the article. Please contact the corresponding author for access to data presented in this study.
